# Bacteriology and mortality of necrotizing fasciitis in a tertiary coastal hospital with comparing risk indicators of methicillin-resistant *Staphylococcus aureus* and *Vibrio vulnificus* infections: a prospective study

**DOI:** 10.1186/s12879-021-06518-5

**Published:** 2021-08-09

**Authors:** Yao-Hung Tsai, Tsung-Yu Huang, Jiun-Liang Chen, Cheng-Ting Hsiao, Liang-Tseng Kuo, Kuo-Chin Huang

**Affiliations:** 1 Department of Orthopaedic Surgery, Chia-Yi Chang Gung Memorial Hospital, 6, West Sec, Chia-Pu Rd., Putz City, Chia-Yi County 613 Taiwan, Republic of China; 2 Division of Infectious Diseases, Department of Internal Medicine, Chia-Yi Chang Gung Memorial Hospital, Putz city, Chia-Yi County Taiwan, Republic of China; 3 College of Medicine, Chang Gung University at Taoyuan, Taoyuan City, Taiwan, Republic of China; 4 Department of Emergency Medicine, Chia-Yi Chang Gung Memorial Hospital, Putz City, Chia-Yi County Taiwan, Republic of China

**Keywords:** Necrotizing fasciitis, *Vibrio vulnificus*, MRSA, Monomicrobial, Gram-negative

## Abstract

**Background:**

*Vibrio vulnificus* has been reported as the leading causative pathogen of necrotizing fasciitis (NF) and related fatality in the coastal area. Necrotizing fasciitis caused by methicillin-resistant *Staphylococcus aureus* (MRSA) and *V. vulnificus* have high mortality rates. The purpose of this prospective study was to clarify the clinical characteristics between death and survival NF patients, to investigate bacteriologic profile and mortality of NF patients, and to compare risk indicators of MRSA and *V. vulnificus* NF patients.

**Methods:**

This prospective study was conducted in 184 consecutive NF patients over a period of three years in a tertiary coastal hospital. Differences in mortality, laboratory findings, microbiology and clinical outcomes were compared between the death and survival groups, and the *V. vulnificus* and MRSA subgroups.

**Results:**

Twenty patients died, resulting in a mortality rate of 10.9%, and there were 108 patients with a monomicrobial infection (58.7%). The death group had a significantly higher incidence of shock at emergency room and bacteremia than did the survival group. *Vibrio* species (40 cases) and *S. aureus* (31 cases) were the two major pathogens. Significant differences with respect to hepatic dysfunction, shock, the event with seawater or seafood contact, bacteremia, C-reactive protein, mean platelet counts, and the Laboratory Risk Indicator for Necrotizing Fasciitis (LRINEC) score were observes between *V. vulnificus* and MRSA groups.

**Conclusions:**

NF patients with both hepatic dysfunction and diabetes mellitus, bacteremia and shock have significantly higher mortality. We should be aware of the increasing incidence of monomicrobial NF and higher mortality rates of Gram-negative pathogens in the warm coastal area. LRINEC score is not a suitable diagnostic indicator for *V. vulnificus* NF, which is more rapidly progressive and fulminant than MRSA NF. NF needed team works by early suspicion, immediate surgical intervention and aggressive care, which can successfully decrease mortality.

## Background

Necrotizing fasciitis (NF) is a life-threatening soft tissue infection with a high mortality rate of 25.3–73% [1–3]. Early suspicion of NF with emergent surgical debridement and appropriate antibiotic therapy can increase the survival rates and clinical outcomes [4, 5]. To improve the NF diagnosis, several clinical features had been recommended in the clinical diagnosis, including bullae, purple skin discoloration, crepitus, gas on X-Ray, local pain, swelling, erythema, tachycardia, fever, hypotension, and tachypnea [6–9]. NF is generally due to external trauma or skin wound that occurs commonly in patients with pre-existing chronic underlying diseases; however, diabetes mellitus, immunosuppression, chronic renal failure and decompensated liver disease have been reported as the major co-morbidities with poor prognosis in NF patients [6–10].

Depending on microbiological findings, NF is classified into four types—Polymicrobial infection (type I) with signs and symptoms of severe septic shock and multiple organ dysfunction was believed to be the first cause of NF, and followed by group A *Streptococcus* infection alone or combined with *Staphylococcus aureus* (type II) [1–10]. However, several studies have found that *S. aureus*, with or without methicillin resistanc, has emerged as an important monomicrobial infection type for NF [6–15]. Meanwhile, the marine bacterial infection related NF, such as *Vibrio species*, *Aeromonas* or *Klebsiella species*, have been noted as type III, and the fungal infection have been classified as type IV [1–7, 15–18].

In a 10-year study from 2003 to 2013, *Streptococcus* (48%), *Staphylococcus* (22%), and Gram-negative bacteria (21%) were the main pathogens to cause death in the patients with NF in the U.S., while monomicrobial NF due to either *Staphylococcus* or *Streptococcus* contributed to 69% of deaths with identified microorganisms [11]. In the past decade, methicillin-resistant *Staphylococcus aureus* (MRSA) was evidenced as an important emerging monomicrobial pathogens with a high mortality rate of up to 15% with a general increasingly range from 4 to 23% [12–15]. Furthermore, MRSA infection has caused a higher amputation rate in patients with deep-seated infection than methicillin-sensitive *Staphylococcus aureus* (MSSA) did [4, 13, 15].

*Vibrio* spp. had been reported as the leading causative pathogen of NF and related fatality in our institution, which is located at warm-water coastal regions in southwest Taiwan [6, 15–18]. *Vibrio* NF usually occurs through the injuries sustained when handling seafood, wound exposure to seawater, and ingestion of contaminated undercooked seafood [15–18]. We have established a treatment strategy including emergency fasciotomy or amputation, antibiotic therapy with a third-generation cephalosporin plus tetracycline, and admission to the intensive care unit (ICU) for patients with fulminant necrotizing fasciitis, such as *Vibrio*, MRSA, and *Aeromonas* infections [6, 15–18]. The Laboratory Risk Indicator for Necrotizing Fasciitis (LRINEC) score has been proposed as a tool to identify patients at a higher risk for NF, in which a LRINEC score of 6 or greater would indicate a high risk for the presence of NF and be widely used [1]. However, our previous study revealed that the LRINEC scoring system is not appropriate for determining early diagnosis with *Vibrio* NF [6]. Our previous study had demonstrated that the clinical features of *Vibrio vulnificus* infection were more rapidly progressive and fulminant than those of the MRSA or MSSA infection, but it was a retrospective study [15]. Thus, we conducted this prospective study to search out the association between LRINEC scores and early detection of *V. vulnificus* and MRSA NF.

The purpose of this prospective study was to determine and clarify the clinical characteristics between dead and survival NF patients. We also investigated microbiological features and the association between mortality and different organisms of the consecutive NF patients, and compared the clinical and laboratory risk indicators of the patients with *V. vulnificus* and MRSA NF on initial examination.

## Methods

### Ethics

This prospective study for the causes and outcomes of patients with surgical confirmed NF of the extremities was approved by the Ethics Committee and Institutional Review Board of Chang Gung Medical Foundation (103-2081B), and conformed to the Declaration of Helsinki. Informed consent was obtained from all subjects included in this study, and all methods were carried out in accordance with relevant guidelines and regulations.

### Patients selection

We included the NF patients who were initially diagnosed by emergency medicine doctors and underwent excisional fasciotomy or immediate limb amputation by orthopedic surgeons admitted to Chia-Yi Chang Gung Memorial Hospital between April 2015 to May 2018. NF was defined by surgically findings: the presence of grayish necrotic soft tissue and hemorrhagic bullaes, loss of resistance of normally adherent fascia to digital blunt dissection, and the appearance of pus with the foul odor of dishwater. The diabetic foot infection and NF patients who did not receive surgery were excluded. A total of the 184 consecutive patients were enrolled into this program. These patients included 120 men and 64 women with a mean age of 66.4 years (range, 19 to 95 years).

### Diagnosis and treatment protocol

**Fig. 1:**
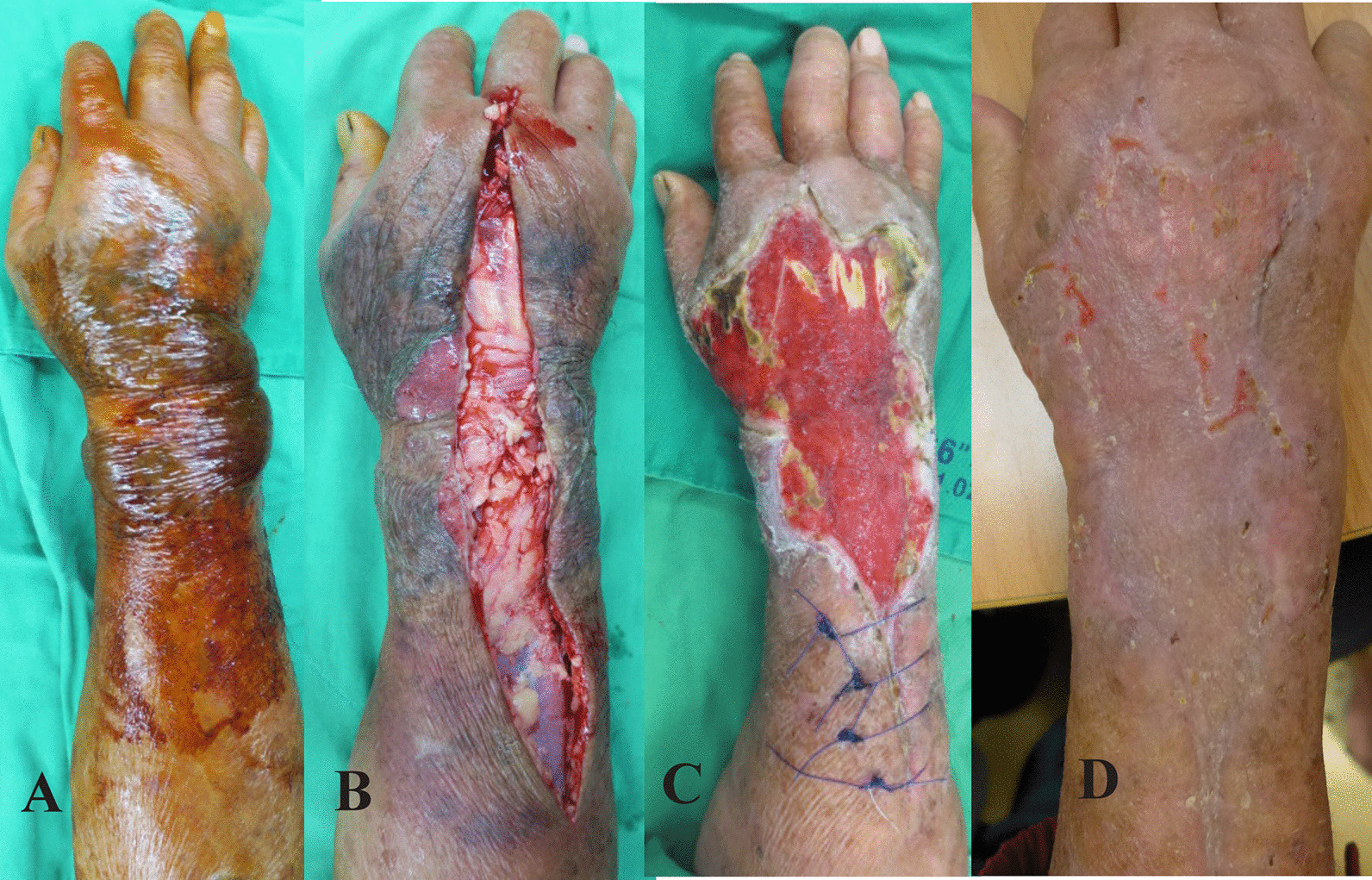
A 73 year-old male fishman with a history of diabete mellitus and oral cancer had right hand pain and swelling for 2 days after handling fish. **A** Preoperative photographs of the right forearm revealed severe patchy purpura, hemorrhagic bullae and edema in the emergency room. **B** After emergency fasciotomy, the forearm revealed extensive necrosis of underlying skin and turbid fascial layer. Three days later, the wound cultures confirmed the presence of *Vibrio vulnificus*. **C** He had received repeated debridement and vacuum assisted closure (VAC) therapy. He had received skin graft on the 42th day after fasciotomy and discharged on the 50th day. **D** He was followed up with a good skin growth of right forearm in the clinic

The most common symptoms of NF patients were pain and swelling of the involved limbs with edematous, patchy, erythematous, and hemorrhagic bullous skin lesions. Contact history with seawater or raw seafood was routinely surveyed. Broad-spectrum antibiotics with usage of ceftriaxone with/without other regimens were initially administered to all the patients, and emergency fasciotomy or immediate limb amputation was performed wherein necrotizing fasciitis was diagnosed at the time of admission to the emergency room (ER) or at the time of consultation in the ward [19]. Surgical debridement was done every other day if progressive necrotic changes were combined with a deteriorating clinical presentation. Initial empiric antibiotics were continued after first surgery and adjusted based on the results of blood cultures and tissue tests a few days later. Hyperbaric oxygen (HBO) therapy and vacuum assisted closure (VAC) therapy was administered in stable NF patients for improving wound healing. Soft tissue reconstructions, such as skin grafts and flap reconstruction, were done until the infected necrotic tissue was controlled and stabilized [6, 16, 19] (Fig. 1).

Demographic and clinical variables of the 184 patients were evaluated and recorded after confirming the diagnosis of NF by histopathological examination. The cultured specimens, obtained from the wounds or the blood, were confirmed by microbiologic evaluation. Identification of these microorganisms was based on standard phenotypic tests used in clinical microbiology laboratories. The specimens of the patients were classified as monomicrobial infection, polymicrobial infection, and no growth, and clinical outcomes were reviewed for each patient according to the microbiological findings. Differences in mortality, chronic illness, related events and clinical courses were compared between the death and survival groups.

### Clinical assessment of *Vibrio vulnificus* and MRSA patients

Further, we enrolled these patients with monomicrobial infection of *V. vulnificus* and MRSA, and categorized them into two subgroups. Differences in age, gender, presence of comorbidities, the interval between contact or injury and admission to ER, the interval between diagnosis and first surgery, affected sites, mortality, laboratory findings at the time of admission, the LRINEC score, and clinical outcomes were compared between the two subgroups.

### Statistical analysis

Statistical analyses were performed with the use of Statistical Product and Service Solutions (SPSS) Version 18.0 statistical software (SPSS, Chicago, Illinois). We used the two-tailed *t*-test for continuous variables and the Fisher exact test for categorical variables to examine significant relationships between risk factors and outcomes between *Vibrio vulnificus* and MRSA groups. A value of *p* < 0.05 was considered significant.

## Results

### Patient characteristics in the death and survival groups

Twenty patients died, resulting in a mortality rate of 10.9%. Thirteen patients had received amputation with an amputation rate of 7%. Sixty-five patients had reported to have a history of seawater or seafood contact, and 28 patients recalled to have a farm injury or a dirty-water contact. The time interval from symptom to presentation at the emergency room ranged from one to four days (mean, 2.2 days) prior to admission. The mean time-interval from admission in the emergency room to the first operation ranged from 2 to 12 h (mean 5.85 h).

Age, gender, interval between symptom and admission, interval between diagnosis of necrotizing fasciitis and first surgery, infective regions, nature of first surgery, and hospital days did not differ significantly between the death and survival groups. Ninety-three patients (50.5%) had associated with a history of hepatic dysfunction, such as liver cirrhosis, hepatitis B or C, hepatocellular carcinoma, or alcoholic liver disease. Seventy-four patients (40.2%) had associated with a history of diabetes mellitus. NF patients with both hepatic dysfunction and diabetes mellitus have higher mortality than did those with hepatic dysfunction or diabetes mellitus alone (*p* = 0.037) (Table 1). The death group had a significantly higher incidence of shock at ER (*p* = 0.0002) and bacteremia (*p* = 0.0004) than did the survival group (Table 2).

**Table 1:** Comparison between the deaths and survivors for charcteristics at first consultation in the ER and ward

		Deaths	Survivors	
		N = 20	N = 164	P value
Age (years)	Mean	66.6	66.1	0.55
Gender (%)				
Male		9 (45)	111 (67.7)	
Female		11 (55)	53 (32.3)	
Interval from contact or injury to presentation at ER (days)	Mean	2.2	3.4	0.32
Interval from diagnosis at ER to first operation (hours)	Mean	4.3	5.8	0.22
Underlying chronic disease (%)				
Hepatic dysfunction and DM		8 (40)	30 (18.3)	0.037*
Hepatic dysfunction alone		7 (35)	48 (29.3)	0.61
Diabetes Mellitus alone or with others		1 (5)	35 (21.3)	0.13
Chronic renal insufficiency		1 (5)	8 (4.9)	
Heart disease		2 (10)	19 (11.6)	
Cancer		1 (5)	8 (4.9)	
Gout		0	4 (2.4)	
Nil		0	12 (7.3)	
Wound location (%)				0.47
Upper extremity		10 (50)	93 (56.7)	
Lower extremity		10 (50)	61 (43.3)	
First operation				0.29
Fasciotomy		19 (95)	162 (98.7)	
Amputation		1 (5)	2 (1.3)	
Final operation after first fasciotomy (%)		19	162	
Amputation		2 (10.5)	8 (4.9)	
Split-thickness skin graft		0	80 (49.4)	
Flap		0	6 (3.7)	
Debridement		8 (42.1)	46 (28.4)	
Without secondary operation		9 (47.4)	22 (13.6)	
Hospital days	Mean	34.25	35.21	0.08

Hepatic dysfunction included liver cirrhosis, hepatitis B or C, hepatocellular carcinoma, and alcoholic liver disease

*Mean p < 0.05 and the difference was significant

**Table 2:** Demographic and clinical presentations of deaths and survivors

Characteristics (%)	Deaths (N-20)	Survivors (N-164)	*p*-value
*Related events*			
Seawater or seafood contact	4 (20)	61 (37.2)	
Farm/dirty-water contact	1 (5)	27 (16.5)	
*Vital signs*			
Body temperature > 38.5 °C	2 (10)	28 (17)	0.53
Heart rate ≥ 100	13 (65)	81 (49.4)	0.24
Respiratory rate > 20	13 (65)	87 (53)	0.35
Systolic blood pressure < 90	10 (50)	20 (12.2)	0.0002*
*Cultures*			
Blood (P) Wound (P)	12 (60)	23 (14)	
Blood (P) Wound (N)	1 (5)	17 (10.4)	
Blood (N) Wound (P)	3 (15)	74 (45.1)	
Blood (N) Wound (N)	4 (20)	50 (30.5)	
Presence of bacteremia	13 (65)	40 (24.4)	0.0004*
*Vibrio vulnificus*	5 (38.5)	22 (55)	
*Aeromonas* spp.	4 (30.7)	5 (12.5)	
*Pseudomonas aeruginosa*	1 (7.7)	1 (2.5)	
*E. coli*	0	1 (2.5)	
MRSA	1 (7.7)	1 (2.5)	
MSSA	0	1 (2.5)	
CONS	0	2 (5)	
β-hemolytic streptococcus	1 (7.7)	6 (15)	
*Viridans streptococci*	0	1 (2.5)	
Polymicrobial	1 (7.7)	0	

Blood (P), culture-positive blood sample; Blood (N), culture-negative blood sample; Wound (P), culture-positive surgical wound sample; Wound (N), culture-negative surgical wound sample; CONS, Coagulase negative *Staphylococcus*

*Mean *p* < 0.05 and the difference was significant

### Microbiological findings in NF patients

Instead of polymicrobial infection, most NF samples were found to have monomicrobial infection (58.7%). Among bacterial species identified, *V. vulnificus* was the most dominant pathogen (21.7%), followed by *S. aureus* (16.8%), β-hemolytic *Streptococcus* (6.5%), *Aeromonas* spp. (6%), and *Pseudomonas aeruginosa* (2.7%). The patients with monomicrobial *Aeromonas* spp and monomicrobial Group non-ABD β-hemolytic *Streptococcus* infections had the highest mortality rate of 45.5 and 33.3% respectively. Fifty-three patients had bacteremia, and *V. vulnificus* patients had a significantly higher incidence of bacteremia (50.9%). Thirteen patients with bacteremia were obtained in the death group, from the blood in one *V. vulnificus* patient and from both blood and wounds in 12 patients. There were four *V. vulnificus*, four *Aeromonas* spp., one *P. aeruginosa*, one MRSA, one Group non-ABD β-hemolytic *Streptococcus* and one polymicrobial specimens revealed positive in both blood and wound cultures. The mortality rate of Gram-negative pathogens was 16.7% (10/60), which was higher than that of Gram-positive pathogens (6.25%, 3/48) (Table 3).

**Table 3:** Bacterial species from necrotizing fasciitis patients

Isolated bacteria	No. of patients (%)	Dead patients	Survival patients	Mortality rate of pathogen (%)
Variable (%)	(N = 184)			
Monomicrobial infection	**108 (58.7%)**	**13**	**95**	**12**
Gram-positive aerobic pathogens	48 (44.4%)	3	45	6.25
* Staphylococcus aureus*	31 (64.6)	2	29	6.5
MRSA	16	1	15	6.3
MSSA	15	1	14	6.7
* Viridans streptococci*	2 (4.2)	0	2	0
Coagulase negative *Staphylococcus*	3 (6.2)	0	3	0
β-hemolytic *streptococcus*	12 (25)	1	11	8.3
Group non-ABD	4	1	3	25
Group B	4	0	4	0
Group A	2	0	2	0
* S. dysgalactiae*	1	0	1	0
* S. equisimilis*	1	0	1	0
Gram-negative aerobic pathogens	60 (55.6%)	10	50	16.7
* Vibrio vulnificus*	40 (66.6)	4	36	10
* Aeromonas* spp	11 (18.3)	5	6	45.5
* Pseudomonas aeruginosa*	5 (8.3)	1	4	20
* Enterobacter cloacae*	1 (1.7)	0	1	0
* E. coli*	1 (1.7)	0	1	0
* Klebsiella pneumoniae*	1 (1.7)	0	1	0
* Shewanella putrefaciens*	1 (1.7)	0	1	0
Polymicrobial infection	**22 (12.0%)**	**3**	**19**	**13.6**
No growth	**54 (29.3%)**	**4**	**50**	**7.4**
Total	**184**	**20**	**164**	**10.9**

Those pathogens identified in the polymicrobial cultures were *S. aureus, Streptococcus* spp., *Escherichia coli*, *Enterobacter cloacae*, *Pseudomonas aeruginosa*, *Enterococcus faecalis*, *Morganella morganii*, *Proteus*, *Peptostreptococcus anaerobius*, *Acinetobacter* spp. and *Bacteroides fragilis*. The mortality rate of polymicrobial NF was 13.6%. Bacterial growth was absent in 54 patients, and 4 patients died, representing a mortality rate of 7.4%.

### Clinical features and laboratory findings difference between MRSA-NF *and V. vulnificus*-NF groups

The mortality rate among the patients infected with MRSA and *V. vulnificus* were 6.25% (1/16) and 10% (4/40), respectively. Age, sex, wound location, vital signs, and outcomes did not differ between the two groups. In the MRSA group, One patient who had liver cirrhosis with diabetes mellitus died. One patient had liver cirrhosis with hepatitis C, one had liver cirrhosis, one had hepatitis C alone, and one had alcoholic liver disease.

In the *V. vulnificus* group, ten patients had hepatitis B or C with diabetes mellitus, and one died. Six patients had liver cirrhosis with/without hepatitis B or C, and one died. One patient who had hepatocellular carcinoma died. Six patients had a history of hepatic hepatitis B and C, and one died. One patients had liver cirrhosis and diabetes mellitus, and one patient alcoholic liver disease. Pre-existing hepatic dysfunction, the interval between contact and admission to ER, hypotensive shock in ER, and the event with seawater or seafood contact were associated with *V. vulnificus* NF. There were 15 V*. vulnificus* NF patients with blood and tissue culture-positive samples, and 11 patients with blood culture-positive samples. Twenty-six patients with *Vibrio*-NF (65%) were found with bacteremia, while only 3 patients with MRSA infection (18.7%) had positive blood and tissue cultures (*p* = 0.002) (Table 4).

**Table 4:** Demographic and clinical presentations of MRSA and *V. vulnificus* associated necrotizing fasciitis patients

Characteristics	MRSA (N-16)	*Vibrio vulnificus* (N-40)	*p*-value
Age (years)			
Mean	62.8	71.2	0.35
Gender			
Female	5	14	0.52
Male	11	26	
Comorbidities (%)			
Hepatic dysfunction	5 (31.2)	25 (62.5)	0.034*
LC alone or with others	3 (18.75)	7 (17.5)	1
Diabetic mellitus	8 (50)	12 (20)	0.136
Cancer	2 (12.5)	2 (5)	0.321
Chronic kidney disease	4 (25)	10 (25)	0.624
Interval from contact or injury	5.5	1.2	0.023*
to presentation at ER (days)			
Interval from diagnosis at ER	5.8	4.7	0.53
to first operation (hours)			
Wound location (%)			
Upper extremity	8 (50)	24 (60)	0.489
Lower extremity	8 (50)	16 (40)	
Outcome	6.25%	10%	
Death	1	4	0.383
Survivors	15	36	
Related events (%)			
Seawater or seafood contact	2 (12.5)	37 (92.5)	0.001*
Farm/dirty-water contacted	1 (6.25)	3 (7.5)	0.43
Vital signs (%)			
Body temperature > 38 °C	4 (25)	8 (20)	0.376
Heart rate ≥ 100	10 (62.5)	22 (55)	0.607
Respiratory rate > 20	7 (43.75)	23 (57.5)	0.325
Systolic blood pressure < 90	0	9 (22.5)	0.036*
Cultures (%)			
Blood (P) Wound (P)	3 (18.75)	15 (37.5)	
Blood (P) Wound (N)	0	11 (27.5)	
Blood (N) Wound (P)	13 (81.25)	14 (35)	
Presence of bacteremia (%)	3 (18.75)	26 (65)	0.002*

Blood (P), culture-positive blood sample; Blood (N), culture-negative blood sample; Wound (P), culture-positive surgical wound sample; Wound (N), culture-negative surgical wound sample

Hepatic dysfunction included liver cirrhosis (LC), hepatitis B or C, hepatocellular carcinoma, and alcoholic liver disease

*Mean *p* < 0.05 and the difference was significant

Analysis of hematology and clinical biochemistry of the NF patients demonstrated no significant difference between these two groups in white blood cell count, segment form neutrophil, band form neutrophil, hemoglobin, albumin, blood sugar, creatinine, and sodium. However, different prevalence patterns between the two groups were observed for C-reactive protein (CRP), mean platelet counts and platelet counts ≤ 1.5 × 10^5^ per mm^3^ (Table 5).

**Table 5:** Blood analysis of MRSA and *V. vulnificus* necrotiz NF patients

		MRSA (N-16)	*Vibrio vulnificus* (N-40)	*p*-value
White cell count (cells/mm^3^)	Mean	16,400 ± 7128	14,560 ± 10,820	0.53
Segmented forms (%)	Mean	79.6 ± 7.3	81.7 ± 10.7	0.47
≦73		2	7	0.494
> 73		14	33	
Band forms (%)	Mean	2.91 ± 5.17	5.54 ± 7.23	0.19
= 0		7	22	0.321
> 0		9	18	
Albumin (g/dL)	Mean	3.47 ± 0.47	3.42 ± 0.46	0.72
< 3.5		9	19	0.384
≥ 3.5		7	21	
Platelet counts (per mm^3^)	Mean	211,200 ± 114,500	139,800 ± 62,940	0.004*
≤ 1.5 × 10^5^		5	26	0.023*
> 1.5 × 10^5^		11	14	
Blood sugar (mg/dL)	Mean	206 ± 110	169 ± 71	0.14
Hemoglobin (g/dL)	Mean	12.3 ± 1.62	13.0 ± 2.11	0.19
Creatinine (mg/dL)	Mean	1.30 ± 0.68	1.52 ± 1.25	0.52
Sodium (mmol/L)	Mean	135 ± 2.2	136 ± 2.6	0.1
C-reactive protein (mg/L)	Mean	184 ± 132	64.5 ± 88.4	0.000*

*Mean *p* < 0.05 and the difference was significant

The LRINEC score showed a significant statistical difference between the two groups (*p* = 0.0003), and the numbers of *V. vulnificus* NF patients with LRINEC score < 6 had a significantly higher than the numbers of patients with MRSA NF group (*p* = 0.004) (Table 6).

**Table 6:** Laboratory risk indicator for necrotizing fasciitis (LRINEC) score of MRSA and *V. vulnificus *necrotiz NF

		Patients		
Variable (%)	Score	*MRSA *(N-16)	*Vibrio vulnificus *(N-40)	*P* value
C-Reactive Protein, mg/L				
< 150	0	8 (50)	34 (85)	0.01*
≧ 150	4	8 (50)	6 (15)	
White cell count, per mm^3^				
< 15	0	8 (50)	24 (60)	
15–25	1	7 (43.75)	13 (32.5)	0.73
> 25	2	1 (6.25)	3 (7.5)	
Hemoglobin, g/dL				
> 13.5	0	3 (18.75)	18 (45)	
11–3.5	1	9 (56.25)	14 (35)	0.175
< 11	2	4 (25)	8 (20)	
Sodium, mmol/L				0.069
≥ 135	0	8 (50)	30 (75)	
< 135	2	8 (50)	10 (25)	
Creatinine, umol/L				
≦ 141	0	12 (75)	32 (80)	0.467
> 141	2	4 (25)	8 (20)	
Glucose, mmol/L				
≦ 10	0	9 (56.25)	26 (65)	0.376
> 10	1	7 (43.75)	14 (35)	
Total score	Mean	6.08 ± 2.86	3.12 ± 2.46	0.0003*
≥ 6		9 (56.25)	6 (15)	0.004*
< 6		7 (43.75)	34 (85)	

*Mean P < 0.05 and the difference was significant

## Discussion

The Chiayi Chang Gung Memorial Hospital is a tertiary hospital which situated on the western coast of southern Taiwan, and *V. vulnificus* are the most frequent causative organism of monomicrobial NF. The residents’ occupations were fishermen or farmers who were frequently associated with handling raw seafood, exposure to seawater, and contact with brackish water or soil [6, 16, 16, 17, 19]. However, *Vibrio* spp. and *Aeromonas* spp. infections have a relatively high incidence and associated with high mortality in our institution [6, 15–17, 19, 20]. We have identified as hypotensive shock, severe hypoalbuminemia, severe thrombocytopenia, and increased banded leukocyte forms can be considered as clinical and laboratory risk indicators to initiate early surgery for *Vibrio* and all types of necrotizing fasciitis [6, 15–17, 19–21]. Moreover, we had established a treatment protocol including emergency fasciotomy or amputation, antibiotic therapy with a third-generation cephalosporin and admission to the intensive care unit for suspected *Vibrio* NF patients, and had successfully decreased the mortality rate of *Vibrio* NF from 35 to 13% during the 6-year period from 2004 to 2010 [6, 15–17, 19, 20]. In this prospective study, we had diminished the mortality rate of total NF to 10.9% and *Vibrio* NF to 10% respectively after early suspicion and aggressive traeatment by the team works.

Diabetes mellitus has been reported to be a common underlying disease in NF patients, accounting for 44–72% in the literatures [1–11]. Many literatures had also demostrated that hepatic dysfunction, such as liver cirrhosis, hepatitis B or C, hepatocellular carcinoma, or alcoholic liver disease, was a highly risk factor for developing NF [1–5, 19, 22–24]. Those virulence factors of micro-organisms, such as *V. vulnificus*, *S. aureus*, β-hemolytic *Streptococcus*, *Aeromonas* spp. and *P. aeruginosa*, were commonly reported to impair the phagocytic activity of the reticuloendothelial system, and to result in bacterial translocation and bacteremia in patients with hepatic decompensation [13–24]. In this study, we found there were 50.5% of NF patients had associated with a history of hepatic dysfunction, and 40.2% had diabetes mellitus. Most of all, those NF patients with both hepatic dysfunction and diabetes mellitus had a significantly higher mortality. Thus, our finding should alert the clinicans to pay more attention and treat aggressively for those NF patients with a history of hepatic dysfunction and/or diabetes mellitus who may result in fulminant clinical course and mortality in a short time.

Recent literatures have revealed that monomicrobial necrotizing fasciitis caused by Gram-negative pathogens, such as *V. vulnificus*, *Klebsiella pneumoniae*, *Aeromonas hydrophila*, *Pseudomonas spp.* and *Escherichia coli*, had persistently increased, and could cause more fulminant clinical courses and higher incidence of mortality rate than Gram-positive pathogens do [19, 25–31]. Monomicrobial Gram-negative NF combined with bloodstream infection was reported to have significantly increased the mortality rate [25–31]. This prospective study indicated that the incidence of monomicrobial necrotizing fasciitis (58.7%) was higher than that of polymicrobial infections (12%). Moreover, we found that the monomicrobial Gram-negative NF patients had a greater proportion (55.6% vs. 44.4%) and revealed higher mortality rates (16.7% vs. 6.25%) than the Gram-positive NF patients. Based on those findings, we should pay more attention to manage Gram-negative NF due to its rapid and fulminant courses with increasing risk of developing bacteremia and poor outcomes.

Our previous study had reported that the clinical characteristics of *V. vulnificus* infection were more rapidly progressive and fulminant than those of the *S. aureus* infection, either MRSA or MSSA [15]. However, since it was a retrospective study, some medical records of patients did not include accurate descriptions and laboratory data. We conducted this prospective study to investigate the outcomes of early detection and surgery of NF, and the association of the specific characteristics and risk factors on initial examination of *V. vulnificus* and MRSA NF. We had demonstrated that *V. vulnificus* NF patients had significant associations with a history of contact with seawater or seafood and MRSA had significant association with a history of diabetes mellitus, previous abrasion injury, pus accumulated in surgical wound, and chronic ulcers [15]. With early diagnosis and emergent surgery, the mortality rates in the patients with *V. vulnificus* and *S. aureus* of 18.3% and 13.1% respectively during the 6-year period from 2003 to 2009 were reduced to a mortality rate of 10% and 6.5% respectively during a 3-year period. Moreover, the mortality rate in the patients with MRSA necrotizing fasciitis was reduced from 17.2 to 6.25% [15].

Liver cirrhosis is considered as a risk factor with increasing mortality among NF patient [2–6, 24, 26, 28, 30]. Seven *Vibrio* NF patients and 3 MRSA NF patients had liver cirrhosis with/without other chronic illness, and one died respectively in this study. We found that one *Vibrio* NF patient (14.3%) and 3 MRSA NF patients (100%) with liver cirrhosis had CRP bigger than 150 mg/L and LRINEC score > 6. Thus, we considered the lower CRP and LRINEC score were related to rapidly progressive and fulminant course of *V. vulnificus* NF, not related to liver cirrhosis.

As a diagnostic tool for severe NF, this prospective study indicated that the application of LRINEC score is species dependent due to the higher score for MRSA group, which may be as a result of longer interval from contact to symptom presentation at ER. We confirmed that the LRINEC scoring system is inappropriate for determining the early diagnosis of *Vibrio* NF patients who revealed fast and fulminant septic status. Otherwise, *V. vulnificus* group had significant differences in the clinical characteristics and laboratory data, such as hypotension at emergency room, shorter interval from contact or injury to symptom presentation at ER, presence of bacteremia, thrombocytopenia, and lower CRP level than MRSA group. These results demonstrated *V. vulnificus* NF revealed more rapidly progressive and fulminant than MRSA NF.

This study has several limitations. First, there was 29.3% of microbial cultures of clinical specimens resulting in negative with the mortality rate of 7.4%. Polymerase chain reaction (PCR) can be considered for early detection and accurate confirmation of the pathogens in sterile sites of these suspicious patients for appropriate antibiotics use. The second limitation was that we did not do LRINEC scoring system for all 184 patients. In fact, our emergency department had reported that the *Vibrio vulnificus* and *Aeromonas hydrophila* NF patients had average LRINEC score of 3.9 and 3.5, which were lower than the mean of the whole NF group. Thus, LRINEC score not be an accurate tool for necrotizing fasciitis risk stratification and differentiation in the suburban and tertiary coastal hospital [32].

## Conclusion

NF patients with both hepatic dysfunction and diabetes mellitus, bacteremia and shock have significantly higher mortality. We should be aware of the increasing incidence of monomicrobial NF and higher mortality rates of Gram-negative pathogens in the warm coastal area. LRINEC score is not a suitable diagnostic indicator for *V. vulnificus* NF, which is more rapidly progressive and fulminant than MRSA NF. NF needed team works by early suspicion, immediate surgical intervention and aggressive care, which can successfully decrease mortality.

## Data Availability

The datasets used and/or analysed during the current study are available from the author (orma2244@adm.cgmh.org.tw) on reasonable request.

## Abbreviations

NF: Necrotizing fasciitis
ER: Emergency room
*S.** aureus*: *Staphylococcus aureus*
*V. vulnificus*: *Vibrio vulnificus*
MRSA: Methicillin-resistant *Staphylococcus aureus*
LRINEC: Laboratory risk indicator for necrotizing fasciitis
ICU: Intensive care unit
